# Bioinformatic analysis of the neprilysin (M13) family of peptidases reveals complex evolutionary and functional relationships

**DOI:** 10.1186/1471-2148-8-16

**Published:** 2008-01-23

**Authors:** Nicholas D Bland, John W Pinney, Josie E Thomas, Anthony J Turner, R Elwyn Isaac

**Affiliations:** 1 Faculty of Biological Sciences, University of Leeds, Leeds, LS2 9JT, UK; 2 Faculty of Life Sciences, University of Manchester, Manchester M13 9PT, UK; 3 INSERM U609, Wellcome Centre for Molecular Parasitology, Glasgow Biomedical Research Facility, 120 University Place, University of Glasgow, Glasgow, G12 8TA, UK

## Abstract

**Background:**

The neprilysin (M13) family of endopeptidases are zinc-metalloenzymes, the majority of which are type II integral membrane proteins. The best characterised of this family is neprilysin, which has important roles in inactivating signalling peptides involved in modulating neuronal activity, blood pressure and the immune system. Other family members include the endothelin converting enzymes (ECE-1 and ECE-2), which are responsible for the final step in the synthesis of potent vasoconstrictor endothelins. The ECEs, as well as neprilysin, are considered valuable therapeutic targets for treating cardiovascular disease. Other members of the M13 family have not been functionally characterised, but are also likely to have biological roles regulating peptide signalling. The recent sequencing of animal genomes has greatly increased the number of M13 family members in protein databases, information which can be used to reveal evolutionary relationships and to gain insight into conserved biological roles.

**Results:**

The phylogenetic analysis successfully resolved vertebrate M13 peptidases into seven classes, one of which appears to be specific to mammals, and insect genes into five functional classes and a series of expansions, which may include inactive peptidases. Nematode genes primarily resolved into groups containing no other taxa, bar the two nematode genes associated with *Drosophila *DmeNEP1 and DmeNEP4. This analysis reconstructed only one relationship between chordate and invertebrate clusters, that of the ECE sub-group and the DmeNEP3 related genes. Analysis of amino acid utilisation in the active site of M13 peptidases reveals a basis for their biochemical properties. A relatively invariant S1' subsite gives the majority of M13 peptidases their strong preference for hydrophobic residues in P1' position. The greater variation in the S2' subsite may be instrumental in determining the specificity of M13 peptidases for their substrates and thus allows M13 peptidases to fulfil a broad range of physiological roles.

**Conclusion:**

The M13 family of peptidases have diversified extensively in all species examined, indicating wide ranging roles in numerous physiological processes. It is predicted that differences in the S2' subsite are fundamental to determining the substrate specificities that facilitate this functional diversity.

## Background

The neprilysin (M13) family of zinc-metallopeptidases is a large group of medically and developmentally important enzymes of which mammalian neprilysin (EC 3.4.24.11, neutral endopeptidase, NEP) was the first member to be biochemically characterised [1]. They metabolise bioactive peptides and are involved in a number of biological processes in mammals including modulation of neurotransmitter levels, reproduction, control of blood pressure and cancer progression. The majority of M13 peptidases described so far are endopeptidases with a strong preference for cleaving the amino-terminal bond of hydrophobic residues [2]. Many are selectively inhibited by phosphoramidon and, typically, the substrates are small to medium sized peptides, including tachykinins, opioid peptides, big-endothelins and bombesin. Recently, it has been shown that two members of the neprilysin family cleave the Alzheimer's amyloid β-peptide (Aβ) in the mammalian brain and that *ex-vivo *expression of neprilysin reduces amyloid plaque burden in a mouse model [3]. Neprilysin and the neprilysin-like peptidases are typically type II integral membrane proteins with their active sites facing the extracellular environment [2]. Soluble neprilysin-like enzymes however do occur in mammalian (human MMEL2 and rodent SEP/NL1 and NEPII) and insect (*Drosophila melanogaster*) tissues [4,5]. Both mammalian and insect soluble enzymes are strongly expressed in the testes suggesting a physiological role in reproduction. Indeed, female mice mated with males lacking SEP/NL1 have smaller litters confirming the important role for this enzyme in mammalian reproduction.

The endothelin converting enzymes (ECE-1 and ECE-2) and the ECE-like group of enzymes all have distinctive biology. ECE-1 exists as four isoforms and has a physiological role in the metabolism of endothelins by generating mature endothelin from the inactive precursor big-endothelin [6]. ECE-1 knockout mice show a fatal developmental phenotype with severe disruption to several developmental processes, including craniofacial development [7]. ECE-2 is predominantly neurally expressed and is thought to be involved in the processing of peptides prior to secretion [8].

ECEL-1 and its rodent homologue, damage-induced neuronal endopeptidase (DINE), are two of the least well-characterised members of the M13 family [9]. DINE was identified due to its up-regulation after neuronal damage and has been shown to be neuro-protective. DINE-knockout mice develop normally, but die immediately after birth due to an inability to inflate their lungs [10]. No physiological substrate for ECEL-1/DINE has been identified, which has hindered understanding the neuro-protective mechanism and its role in respiratory control.

PHEX is a gene that has been shown to be deficient in patients suffering X-linked hypophosphataemic rickets (XLH) [11]. PHEX has no known natural substrate, although it has been reported that it may cleave FGF-23, a member of the fibroblast growth factor family that inhibits renal tubular phosphate transport [12]. PHEX is unusual for an M13 peptidase in that it appears to have a preference for acidic residues at its S1' site [13]. Kell is an important blood group antigen but is also found in the sertoli cells in the testes [14,15]. Kell can convert big endothelin-3 to biologically active endothelin-3 from its precursor protein [14]. Kell is an atypical M13 peptidase having no transmembrane domain, but is instead normally covalently anchored to the membrane protein XK [16]. Deficiency in Kell does not cause disease, but XK null patients (who also lack Kell) suffer from McLeod's syndrome, which leads to acanthocytic anaemia [16].

Invertebrate M13 peptidases have been found in organisms ranging from *Hydra vulgaris *through to highly derived insects such as *Drosophila melanogaster*. The sea snail *Aplysia californica *and the mussels, *Mytilus edulis *and *Mytilus galloprovincialis*, have neprilysin-like peptidases [2]. These activities are involved in control of feeding in the snail and have been implicated in modulating the response of mussel immune effector cells [17,18]. Free-living and parasitic nematodes also possess neprilysin-like peptidases that cleave peptide bonds N-terminal to hydrophobic residues and are inhibited by phosphoramidon [19-22]. A role for neprilysin in nematode locomotion and reproduction has been established in a study of a deletion mutant of *Caenorhabditis elegans *NEP1 [23]. Neprilysin-like activity is enriched in the brain neuropil and in isolated synaptic membranes of relatively basal insects, the locusts,*Schistocerca gregaria *and *Locusta migratoria *and the cockroach *Leucophea maderae*, indicating an evolutionarily conserved role for M13 peptidases in the functioning of nervous systems that use neuropeptides extensively as neurotransmitters/modulators [2,24]. Recently an ECE-like gene was identified in *L. migratoria *and was shown to be highly expressed in the central nervous system and the midgut [25]. Insect M13 peptidases are associated with metamorphosis [26,27] and immunity to bacterial, fungal and protozoan infections [28,29]. The most thoroughly characterised insect M13 peptidase is *D. melanogaster *NEP2, which is expressed in the stellate cells of Malpighian (renal) tubules and in the testes of adult male flies [4,30], where expression is strongest in the elongating cyst cells.

Genome sequencing projects and individual gene studies have provided a large and expanding set of protein sequence data for comparative genomic and phylogenetic studies. Phylogenetics has traditionally concentrated on deducing the evolutionary relationships of various taxa being examined, ranging from whole reconstruction of phyla level relationships to finer resolution studies of individual species groups. However, phylogenetic techniques can also be used to attempt to unravel the functional and evolutionary relationships of a set of paralogous genes. The work presented here provides an extensive examination of the functional relationships of M13 peptidases and provides new insights into the evolution of this medically important family of peptidases.

## Results and Discussion

### Identification and alignment of M13 protein sequences

A combination of methods (including, BLASTP, PSI-BLAST and HMMs) were employed to identify M13 sequences from the genomes of seventeen organisms, eleven of which were from fully sequenced genomes and six of which were from individual entries in GenBank (Additional file 1). Simple BLASTP analysis against NCBI, ENSEMBL and various species specific databases identified the majority of sequences used in this study (Additional file 1); human neprilysin [NP_009220] was used as the query sequence. The remainder were identified from the raw genomic data of *D. pseudoobscura *and *Ap. mellifera *using the SHARKhunt software [31], This was particularly valuable for the analysis of the *Ap. mellifera *genome, as we identified two unannotated genes (Ame1 and Ame6).

**Figure 1:**
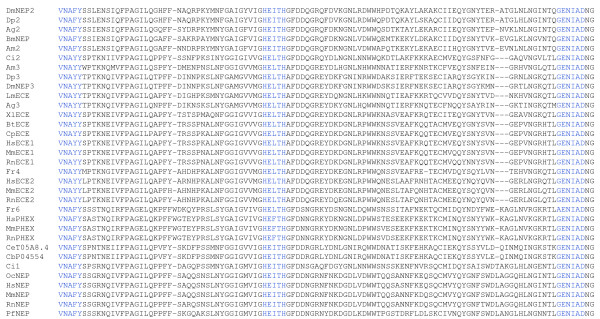
**A section of a multiple sequence alignment of M13 peptidases**. A multiple sequence alignment of 111 protein sequences was generated using MUSCLE [32,33] and was as the basis for the analysis presented. A highly conserved section of the alignment, representing residues 541 to 652 of human neprilysin, contains important catalytic residues. These residues include the HExxH zinc binding motif and the catalytically important GENIAD and VNAFY motifs which are coloured blue. For full alignment see additional file 2.

The large dataset of protein sequences described above was used to produce a multiple sequence alignment (Figure 1, Additional file 2). The program, MUSCLE was used to align these sequences [32,33]. This program was chosen as it aligns areas of strong local homology, which is advantageous because of the strong conservation of catalytic regions, but high variability in other regions of M13 proteins, in particular, the N-terminal region. This alignment was edited to remove large uninformative insertions and was then analysed manually to determine the conservation of key catalytic residues (Figure 1) as defined by mutagenesis studies and by the crystal structure of human neprilysin [34,35].

### Phylogenetic analysis

**Figure 2:**
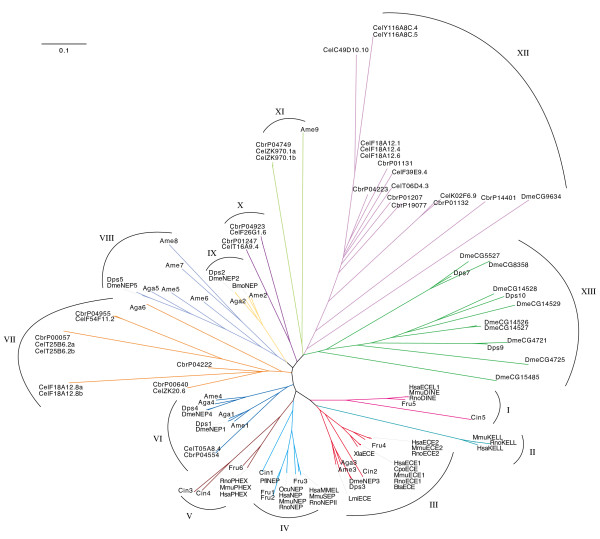
**Phylogenetic analysis of M13 peptidases**. Majority consensus tree of all three methods of phylogenetic reconstruction. The tree was generated using CTree [65]. See additional file 3 for bootstrap values. Key to species; Dme *Drosophila melanogaster*, Aga *Anopheles gambiae*, Dps *Drosophila pseudoobscura*, Lmi *Locusta migratoria*, Bmo *Bombyx mori*, Ame *Apis mellifera*, Hsa *Homo sapiens*, Rno *Rattus norvegicus*, Mmu *Mus musculus*, Fru *Fugu rubripes*, Pfl *Perca flavescens*, Xla *Xenopus laevus*, Ocu *Oryctolagus cuniculus*, Bta *Bos taurus*, Cpo *Cavia porcelllus*, Cin *Ciona intestinalis*, Cel *Caenorhabditis elegans and *Cbr *Caenorhabditis briggsae*.

Three methods of phylogenetic reconstruction were compared in this study and were resolved into a single consensus tree (Figure 2; for bootstrap values see Additional file 3). The three methods were able to resolve clades at the extremes of the branches of the tree with a strong consistency across all three methods. Deeper resolution was less complete with many branches originating from the same node (Figure 2).

### Insect neprilysins form five main clades

Insect neprilysins from *An. gambiae*, *Ap. mellifera *and *D. pseudoobscura *cluster in five groups of strongly conserved genes that delineate with the five *D. melanogaster *genes that show the greatest similarity to human neprilysin (Figure 2 III, VI, VIII, IX). These five clades are present in all phylogenetic reconstructions and each *D. melanogaster *gene can be seen to have a *D. pseudoobscura, An. gambiae*, and *Ap. mellifera *homologue, indicating that these genes evolved before the divergence of the Hymenoptera and the Diptera, approximately 35 and 260 million years ago, respectively [36,37].

The majority of the five clades described above contain only insect genes. Interestingly, several insect NEP genes, including DmeNEP1 and DmeNEP4, form a cluster with the *C. elegans *gene CelTO5A8.4 and its *C. briggsae *homologue CbrPO4554 (Figure 2 VI). DmeNEP1, in particular, also shares an extremely high conservation of active site residues with CelTO5A8.4, suggesting they are functionally very similar. This is the only example of *Drosophila *genes clustering with nematode genes. Neighbour-joining places CelTO5A8.4 and CbrPO4554 outside the DmeNEP1/DmeNEP4 cluster. However, maximum likelihood places them within the cluster with a strong relationship to DmeNEP1. Therefore, it is unclear what is the true position of these nematode genes within this cluster and whether DmeNEP1 and DmeNEP4 were formed from a duplication before or after divergence of the Nematoda and Insecta

DmeNEP3 contains the ECE-like VNAYY motif (Figure 1) and was seen to cluster with the extended clade containing the ECE sub-group of enzymes (Figure 2 III). This is the only example of insect and vertebrate genes clustering in this analysis and is seen with all three types of analyses. There is however some variation as to the position of the *C. intestinalis *gene Cin2. *C. intestinalis *is a urochordate and shares a very ancient common ancestor with vertebrates, having diverged from that lineage before the evolution of the Craniata and therefore provides the best insight into an intermediate between vertebrates and invertebrates [38]. The neighbour-joining method places Cin2 outside the cluster of insect and vertebrate genes. Character based methods however, return trees which better reflect the evolutionary relationships of these species with Cin2 inside the clade between the insect and vertebrate clusters (Figure 2 III). It seems reasonable to assume that Cin2 evolved from the same ancestral ECE from which the vertebrate ECEs also evolved. This provides strong evidence that these genes arose early in the metazoa before the evolution of the majority of organisms used in this analysis.

DmeNEP2 is a soluble secreted enzyme found in the renal tubules and testes of *D. melanogaster*. Interestingly, the mammalian group of soluble peptidases (MmuSEP and HsaMMELII) appears to have evolved recently (Figure 2 IV), after the split from the urochordates and is therefore un-related to DmeNEP2. DmeNEP2 expression is modulated by dietary phosphate, leading to the suggestion that this enzyme is functionally related to HsaPHEX [39]. However, the fly enzyme lacks the highly conserved PHEX motifs [40] and presumably does not share the preference of HsaPHEX for acidic residues in the P1' position [4,12,30]. Therefore, it is not surprising that in the current analysis DmeNEP2 shows no association with the PHEX cluster.

### Vertebrate M13 genes form distinct functionally related clades

The majority of the vertebrate M13 peptidases delineate into functionally related clusters representing the PHEX, Kell, ECE-1, ECE-2, ECEL1, neprilysin and MMELLII groups of peptidases (Figure 2 I-V). All of these clusters contain human and rodent sequences, with the majority also containing *Fugu rubripes *NEPs. Most of these groups have one or more *C. intestinalis *sequence forming a root at the base of the cluster, indicating that these genes evolved before the divergence of these lineages. The Kell group of genes has neither a *C. intestinalis *nor a *F. rubripes *sequence associated with it, suggesting that Kell is a recently evolved member of the M13 family, restricted to mammals (Figure 2 II). A single *C. intestinalis *sequence roots the clade containing NEP/MMELLII clusters (Figure 2 IV). As mentioned previously, this implies that the neprilysin and soluble members of the family arose during the evolution of vertebrates. Both clusters contain fish sequences indicating that, although these sequences evolved after the evolution of the Craniata, they must have arisen early in vertebrate evolution. There are two *F. rubripes *sequences which cluster with neprilysin, indicating either a gene duplication event in fish, or a gene loss in mammals. Similarly, it appears that ECE-1 and ECE-2 arose from a common ancestral gene after the divergence of the vertebrates and urochordates. Interestingly, no *F. rubripes *sequence is found in the ECE-1 sub-cluster (Figure 2 III), which may indicate that ECE-2 is the prototypical member of this group as ECE-2 is seen to cluster with a *F. rubripes *sequence. Though, it is possible that *F. rubripes *may have lost an ECE-1 gene, the drastic consequences of losing ECE-1 in mammals makes this unlikely. Also the genome of another fish, *Tetraodon nigroviridis*, contains two ECE-like genes and the frog, *Xenopus laevis*, has an ECE-1 protein, and therefore it is conceivable that the ECE-1 sequence of *F. rubripes *has yet to be determined. Maximum likelihood analysis suggests the possibility that Kell is derived from the ECE subgroup, which is consistent with the report that Kell efficiently cleaves big-endothelin-3, although the physiological relevance of this *in vitro *study is unclear [16]. The placing of Kell, however, may simply be due to its unusual characteristics masking true evolutionary relationships.

Two *C. intestinalis *(Cin3 and Cin4) genes are seen to cluster with the PHEX group of peptidases indicating a potential recent, gene duplication in *Ciona *(Figure 2 V). The predicted proteins are very similar to each other with only minor changes to the side chains that form the substrate binding pockets, suggesting possible substrate specificity differences.

### Invertebrate gene expansions

The majority of the nematode genes analysed in this work form a series of extended clades (Figure 2 VII, X-XII). The majority of *C. elegans *genes cluster with a *C. briggsae *homologue indicating that these gene expansions arose before these species split, approximately 100 million years ago (mya) [41]. However some species-specific expansions have also occurred, consistent with these families continuing to evolve in these lineages. The most thoroughly described *C. elegans *M13 peptidase ZK20.6 (NEP-1) clusters with several worm peptidases, but is quite distinct from sequences from outside the Nematoda.

Outside of the five clades described above, the majority of *Drosophila *sequences form a series of *Drosophila*-specific expansions (Figure 2 XIII). Many of these expansions contain at least one *D. pseudoobscura *sequence again suggesting that the expansion occurred before the divergence of these species approximately 35 mya [37]. The expansion of the NEP family is larger in *D. melanogaster*, which may have resulted from different selection pressures on these species. These expansions appear to have occurred fairly early in the diversification of the old world *Drosophila *as *D. melanogaster *and *D. pseudoobscura *represent two of the major species complexes of the *Drosophila *genus. The fact that the clusters containing these expansions contain no *An. gambiae *or *Ap. mellifera *sequences is confirmatory evidence for these expansions being specific to *Drosophila *and not due to gene loss in other organisms. All of the genes belonging to these groups are expressed (as full length ESTs), but some lack key catalytic residues, indicating they probably encode catalytically inactive proteins with possibly novel non-peptidase roles. Non-enzymatic functions for metallopeptidase-like proteins have been shown to be important for development in other organisms [42-44], but there is currently no evidence for physiological roles for these non-peptidase members of the M13 family. Nevertheless, the fact that these proteins have been conserved as non-peptidases over 35 million years of evolution suggests that they do indeed have important functions.

### Conservation of catalytically and structurally important residues

Site-directed mutagenesis studies together with the elucidation of the crystal structure of human neprilysin complexed with specific inhibitors have led to a detailed understanding of the structure of the active site and the catalytic mechanism [34]. In human neprilysin, this involves co-ordination of the zinc ion by His^583^, His^587 ^and Glu^646^, and the involvement of Glu^584 ^in polarising the water molecule that attacks the peptide bond of the substrate. These residues and the substrate co-ordinating asparagine (Asn^542^) were generally conserved across the protein sequences examined, apart from the aforementioned invertebrate non-peptidases (Figure 1, Additional file 2).

There is also good conservation of the cysteine residues involved in the formation of the disulphide bonds of neprilysin which is indicative of a general conservation of the tertiary structure of M13 peptidases. However, a cysteine bridge between Cys^233 ^and Cys^241 ^of human neprilysin is only present in mammalian neprilysins and no other M13 peptidases (Additional file 2). The absence of this disulphide bond in HsaECE-1 and RnoNEPII shows that it is not essential for M13 peptidase activity *per se*. Several genes code for proteins that also lack an equivalent to Cys^142 ^of human neprilysin that forms a bridge with Cys^410^. Interestingly, this bond is also not required for peptidase activity of DmeNEP2, which lacks an equivalent to Cys^142 ^[4]. Conservation of Cys^410 ^is much more common, which may suggest an alternative disulphide bridge or function for this residue in other proteins. Further structural data on M13 peptidases will hopefully resolve these issues. Thirty sequences had significant deletions or substitutions in important catalytic or structural positions and were all invertebrate sequences except for *Rattus norvegicus *Kell, which has a lysine instead of the water-activating Glu^584 ^and a serine rather than the zinc co-ordinating Glu^646 ^of human neprilysin (Additional file 2).

**Figure 3:**
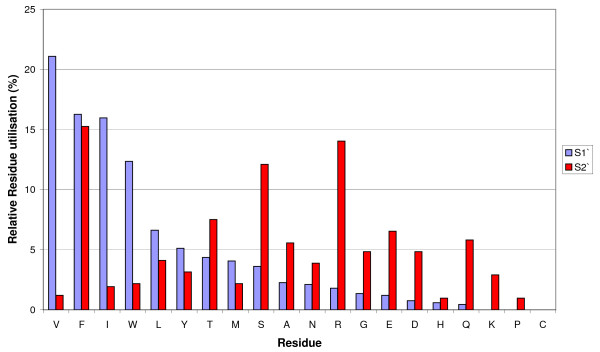
**Utilisation of residues by ligand binding subsites of M13 peptidases**. The utilisation of the twenty amino acids in both the S1' and S2' subsites was examined. The percentage contribution of each amino acid to either binding site was calculated and residues were placed in the order of descending frequency for the S1' subsite.

Of particular importance in determining cleavage site specificity are the S1' and S2' sub-sites that interact with the side-chains of the two residues immediately C-terminal to the scissile peptide bond. Multiple alignment of the neprilysin sequences indicated that there was a greater degree of variation in the S2' than in the S1' subsite (Figure 3). The majority of S1' substitutions are relatively conservative, maintaining a hydrophobic environment (Figure 3). The S1' pocket of the *Ap. mellifera *protein Ame7 also contains a glutamate for isoleucine substitution, as well as the substitution of tyrosine and serine for the hydrophobic phenylalanine and valine, respectively resulting in a pocket that would presumably be more accommodating for charged or polar residues.

The S2' subsite shows a greater spectrum of properties, from being predominantly charged, as in human neprilysin, to being predominantly polar as in *D. melanogaster *NEP2, to being almost entirely hydrophobic as in *Ap. mellifera *Ame4. Interestingly, the S2' subsite of PHEX is highly polar, which is consistent with PHEX's strong P2' preference for polar residues [12]. The conservation of a hydrophobic S1' subsite provides a likely explanation why M13 peptidases from distantly related organisms retain the ability to cleave similar substrates, whereas the range of side chains found at the S2' subsite might determine the different peptide bond preferences seen between enzymes [2,4,25,30]. Variation in the properties of the S2' subsite may be particularly informative about evolutionary pressures on diversifying the physiological substrates of these peptidases [4,45].

The ^541^VNAFY^545 ^motif of human neprilysin is important for the orientation of the peptide bond between the P1 and P1' positions of the substrate [34]. This motif is conserved, although there is some variation which is frequently associated with specific functional classes. The ECE subgroup of enzymes have VNAYY rather than the VNAFY motif [25,46], which is important for determining the specificity of ECE-1 for big-endothelin [6]. Interestingly, the ECE-like group of enzymes (ECEL-1, DINE, etc), for which there are no known physiological substrates, are unique in that all have a LNAYY motif, which may influence substrate specificity. The VNAYY motif is present in a number of other sequences including the Kell proteins as well as various insect M13 peptidases. Both DmeNEP3 and DmeNEP4 contain the VNAYY motif, whereas DmeNEP1 and DmeNEP2 contain the neprilysin-like VNAFY. All the sequences that cluster with DmeNEP5, apart from Ame6, contain a unique version of the VNAFY motif that has a histidine in place of the tyrosine. The tyrosine in the VNAFY motif of human neprilysin is part of a hydrogen bonding network, which is conserved in other M13 peptidases [46,47]. It is not clear what effect this substitution or some of the other substitutions described here would have; however it is likely that structural changes occur, with possible knock-on effects on the properties of the enzymes.

The GENIAD motif contains the zinc-co-ordinating glutamate and is generally conserved amongst the M13 peptidases (Figure 1). In some of the more divergent sequences significant variation is seen in this motif and will probably indicate a loss of activity, as residues in this sequence have been shown to be critical for endopeptidase activity [35]. However, some sequences show small changes which may not disrupt peptidase activity. For example, the majority of *F. rubripes *genes contain a glycine to alanine substitution producing an AENIAD consensus. Interestingly the Kell proteins have a distinct motif (LENAAD) that is conserved across all Kell proteins examined. All substitutions in this motif are conservative, with similar overall side chain properties.

The analysis presented here highlights the functional diversity and complicated evolutionary relationships of M13 peptidases. The phylogenetic analysis successfully resolved vertebrate M13 peptidases into seven classes, one of which appears to be specific to mammals, and insect genes into five functional classes and a series of expansions, which may include inactive peptidases. Nematode genes primarily resolved into groups containing no other taxa, bar the two nematode genes associated with DmeNEP1 and DmeNEP4. This analysis reconstructed only one relationship between chordate and invertebrate clusters, that of the ECE sub-group and the DmeNEP3 related genes. This may be because sequences have diverged so far that reconstructing an evolutionary relationship is impossible. Evolution of these classes could be quite cryptic due to the high rate of evolution and gene loss in the invertebrates [48-51]. If this is the case then it is possible that these proteins are also no longer functionally similar. However, as the S1' and S2' subsites are the main factors in defining M13 substrate specificity, active site analysis may still give insights into functional similarities.

## Conclusion

Our analysis shows the M13 family of peptidases to have diversified extensively in all species examined, indicating wide ranging roles in numerous physiological processes. It is predicted that differences in the S2' subsite are fundamental to determining the substrate specificities that facilitate this functional diversity. The work presented here provides the most thorough and sophisticated analysis to date of the phylogenetic relationships of these peptidases and will provide a strong framework for the study of these genes across model systems and in humans.

## Methods

### *In silico *identification of M13 genes

M13 gene sequences were identified by probing sequence repositories using the BLASTP program [52]. Human neprilysin [NP_009220] was used as the query sequence. Protein sequences from: *Drosophila melanogaster*, *Caenorhabditis briggsae, C. elegans*, *Anopheles gambiae*, *Ciona intestinalis*, *Fugu rubripes*, *Mus musculus*, and *Rattus norvegicus *were obtained from NCBI, ENSEMBL and species specific databases (Additional file 1) [53-58]. Human and all other mammalian sequences were identified by the BLASTP program from the sequence data at NCBI. Rat Kell was produced by concatenating the two partial sequences found in the *R. norvegicus *genome. M13 genes from *D. pseudoobscura *and *Ap. mellifera *were identified from raw genomic data using the SHARKhunt program [31]. SHARKhunt uses a search protocol that combines PSI-BLAST with profile Hidden Markov Model (HMM) techniques. The gene model employed was generated from fifteen M13 protein sequences from across the taxa examined here (Additional file 1). Some *Apis mellifera *sequences were also obtained from annotated entries in NCBI (Additional file 1).

### Phylogenetic reconstruction of M13 genes

Protein sequences determined using the methods described above, were aligned using the program MUSCLE with default settings [32,33]. To facilitate analysis, this alignment was edited using BioEdit software [59] to remove gaps and uninformative insertions. Three methods were used to reconstruct phylogenetic relationships of 111 NEP-like proteins. These were: neighbour-joining, maximum parsimony and maximum likelihood. Neighbour-joining [60] analysis was performed on the alignment described above using PAUP 4.0 [61] and was set to bootstrap the tree 1000 times (Additional file 4). Maximum parsimony analysis was performed using PAUP 4.0 [61]. Trees were generated by the random addition (ten replicates) of sequences from the alignment described above. After completion of trees, branches were swapped using the "tree-bisection and reconnection" method and the most parsimonious trees were saved. From this a consensus tree was generated and bootstrapped 1000 times (Additional file 5). Maximum likelihood analysis was carried out using the PROML function of PHYLIP [62]. Trees were generated using the PAM250 substitution score matrix and a gamma distribution of 1.78 as determined using ProtTest [63]. This model was used to generate 100 bootstrap replicates from which a consensus tree was generated. This tree was re-rooted using NOTUNG [64] to produce a tree that more closely resembled distance and parsimony trees (Additional file 6). These trees were used to generate a majority consensus tree using the consense function of PHYLIP [62]. For clarity an unrooted circular tree omitting bootstrap values (Figure 2) was produced using CTree and a rectangular cladogram incorporating bootstrap values was also produced (Additional file 3) [65].

### Site-specific analysis of M13 sequences

Specific sites in M13 protein sequences were compared using the multiple sequence alignment described above. Sites of interest comprised residues previously identified as being important for catalysis and substrate binding by site-directed mutagenesis studies and from the crystal structure of human neprilysin [34,35]. In the absence of structural data, only residues directly aligned with catalytically important regions of human neprilysin were considered and not adjacent residues that may form portions of binding sites in other enzymes.

## Authors' contributions

NB, JP and JET carried out the analyses. NB, AJT and REI planned the study and drafted the manuscript. All authors read and approved the final manuscript.

## Supplementary Material

Additional file 1 Proteins used in this study. A comprehensive list of all proteins used in this study including accession numbers, source of the sequence and abbreviations used in the manuscript. Underlined sequences were used to generate the SHARKhunt gene model. Click here for file

Additional file 2 Multiple sequence alignment of M13 protein sequences. A multiple sequence alignment of M13 proteins generated using MUSCLE and edited to remove uninformative regions. Click here for file

Additional file 3 Consensus tree of phylogenetic analyses of M13 peptidases. A majority consensus cladogram of three methods of phylogenetic reconstruction of M13 proteins, including percentage bootstrap values in the order: neighbour-joining/maximum parsimony/maximum likelihood. Click here for file

Additional file 4 Neighbour-joining analysis of M13 peptidases. Cladogram of neighbour-joining reconstruction of M13 proteins, including percentage bootstrap values. Click here for file

Additional file 5 Maximum parsimony analysis of M13 peptidases. Cladogram of maximum parsimony reconstruction of M13 proteins, including percentage bootstrap values. Click here for file

Additional file 6 Maximum likelihood analysis of M13 peptidases. Cladogram of maximum likelihood reconstruction of M13 proteins, including percentage bootstrap values. Click here for file
