# Supplementary material for: Bioinformatic analysis of the neprilysin (M13) family of peptidases reveals complex evolutionary and functional relationships
Source: BMC Evol Biol. 2008 Jan 23;8:16. doi: 10.1186/1471-2148-8-16 (PMC2259306; doi:10.1186/1471-2148-8-16)
Supplement: Additional file 1 — Proteins used in this study. A comprehensive list of all proteins used in this study including accession numbers, source of the sequence and abbreviations used in the manuscript. Underlined sequences were used to generate the SHARKhunt gene model. [file 1471-2148-8-16-S1.pdf]

Name	Accession number	Organism	Source
DmeNEP1	CG5905	*Drosophila melanogaster*	FlyBase
DmeNEP2	CG9761		
DmeNEP3	CG9565		
DmeNEP4	CG4058		
DmeNEP5	CG6265		
DmeCG14526	CG14526		
DmeCG5527	CG5527		
DmeCG14527	CG14527		
DmeCG14528	CG14528		
DmeCG15485	CG15485		
DmeCG14529	CG14529		
DmeCG8358	CG8358		
DmeCG4721	CG4721		
DmeCG4725	CG4725		
DmeCG9634	CG9634		
Aga1	XP 318885<br>ENSANGP00000015756	*Anopheles gambiae*	ENSEMBL
Aga3	XP 317711<br>ENSANGP00000010035		
Aga2	XP 321277<br>ENSANGP00000008439		
Aga4	XP 312790<br>ENSANGP00000003181		
Aga5	XP 312504<br>ENSANGP00000001161		
Aga6	XP 306840<br>ENSANGP00000000184		
Dps1		*Drosophila pseudoobscura*	This study
Dps2			
Dps3			
Dps4			
Dps5			
Dps7			
Dps9			
Dps10			
LmiECE	AAN73018	*Locusta migratoria*	NCBI
BmoNEP	BAB33300	*Bombyx mori*	
Ame1		*Apis mellifera*	This study

Ame2	XP 392043		NCBI
Ame3	XP 393860		
Ame4	XP 394870		
Ame5	XP 394794		
Ame6			This study
Ame7	XP 394512		NCBI
Ame8	XP 393860		
Ame9	XP 393242		
HsaNEP	NP 009220	*Homo sapiens*	
HsaMMEL2	NP 258428		
HsaECE1	NP 001388		
HsaECE2	NP 055508		
HsaPHEX	NP 000435		
HsaECEL1	NP 004817		
HsaKELL	NP 000411		
RnoNEP	NP 036740	*Rattus norvegicus*	
RnoNEPII	XP 233712		
RnoECE1	NP 446048		
RnoECE2	NP 001002815		
RnoPHEX	NP 037136		
RnoDINE	NP 068544		
RnoKELL	XP 342675 XP 216130		
MmuNEP	NP 032630	*Mus musculus*	
MmuSEP	NP 038811		
MmuECE1	NP 955011		
MmuECE2	NP 647454		
MmuPHEX	NP 035207		
MmuDINE	NP 067281		
MmuKELL	NP 115929		
Fru1	lcl|SINFRUUP00000050651	*Fugu rubripes*	ENSEMBL
Fru2	lcl|SINFRUUP00000050652		
Fru3	lcl|SINFRUUP00000076626		
Fru4	lcl|SINFRUUP00000061415		
Fru5	lcl|SINFRUUP00000079115		
Fru6	lcl|SINFRUUP00000073981		
PflNEP	AAC28366	*Perca flavescens*	NCBI
XlaECE	AAH46653	*Xenopus laevus*	
OcuNEP	P08049	*Oryctolagus cuniculus*	
BtaECE	AAB32062	*Bos taurus*	

CpoECE	AAB46734	*Cavia porcelllus*	
Cin1	Ci0100146342	*Ciona intestinalis*	JGI
Cin2	Ci0100130428		
Cin3	Ci0100143599		
Cin4	Ci0100130465		
Cin5	Ci0100139058		
CelT05A8.4	T05A8.4	*Caenorhabditis elegans*	WormBase
CelF18A12.8a	F18A12.8a		
Cel10ZK20.6	10ZK20.6		
CelF18A12.8b	F18A12.8b		
CelT16A9.4	T16A9.4		
CelF26G1.6	F26G1.6		
CelF54F11.2	F54F11.2		
CelZK970.1a	ZK970.1a		
CelZK970.1b	ZK970.1b		
CelC49D10.10	C49D10.10		
CelF18A12.1	F18A12.1		
CelT25B6.2b	T25B6.2b		
CelT25B6.2a	T25B6.2a		
CelT06D4.3	T06D4.3		
CelF39E9.4	F39E9.4		
CelF18A12.6	F18A12.6		
CelK02F6.9	K02F6.9		
CelY116A8C.4	Y116A8C.4		
CelF18A12.4	F18A12.4		
CelY116A8C.5	Y116A8C.5		
CbrP04554	P04554	*Caenorhabditis briggsae*	
CbrP04222	P04222		
CbrP00640	P00640		
CbrP01247	P01247		
CbrP04923	P04923		
CbrP04955	P04955		
CbrP04749	P04749		
CbrP04223	P04223		
CbrP01131	P01131		
CbrP00057	P00057		
CbrP19077	P19077		
CbrP01207	P01207		
CbrP01132	P01132		

CbrP14401	P14401		
