# Supplementary figures and images for: Bioinformatic analysis of the neprilysin (M13) family of peptidases reveals complex evolutionary and functional relationships
Source: BMC Evol Biol. 2008 Jan 23;8:16. doi: 10.1186/1471-2148-8-16 (PMC2259306; doi:10.1186/1471-2148-8-16)

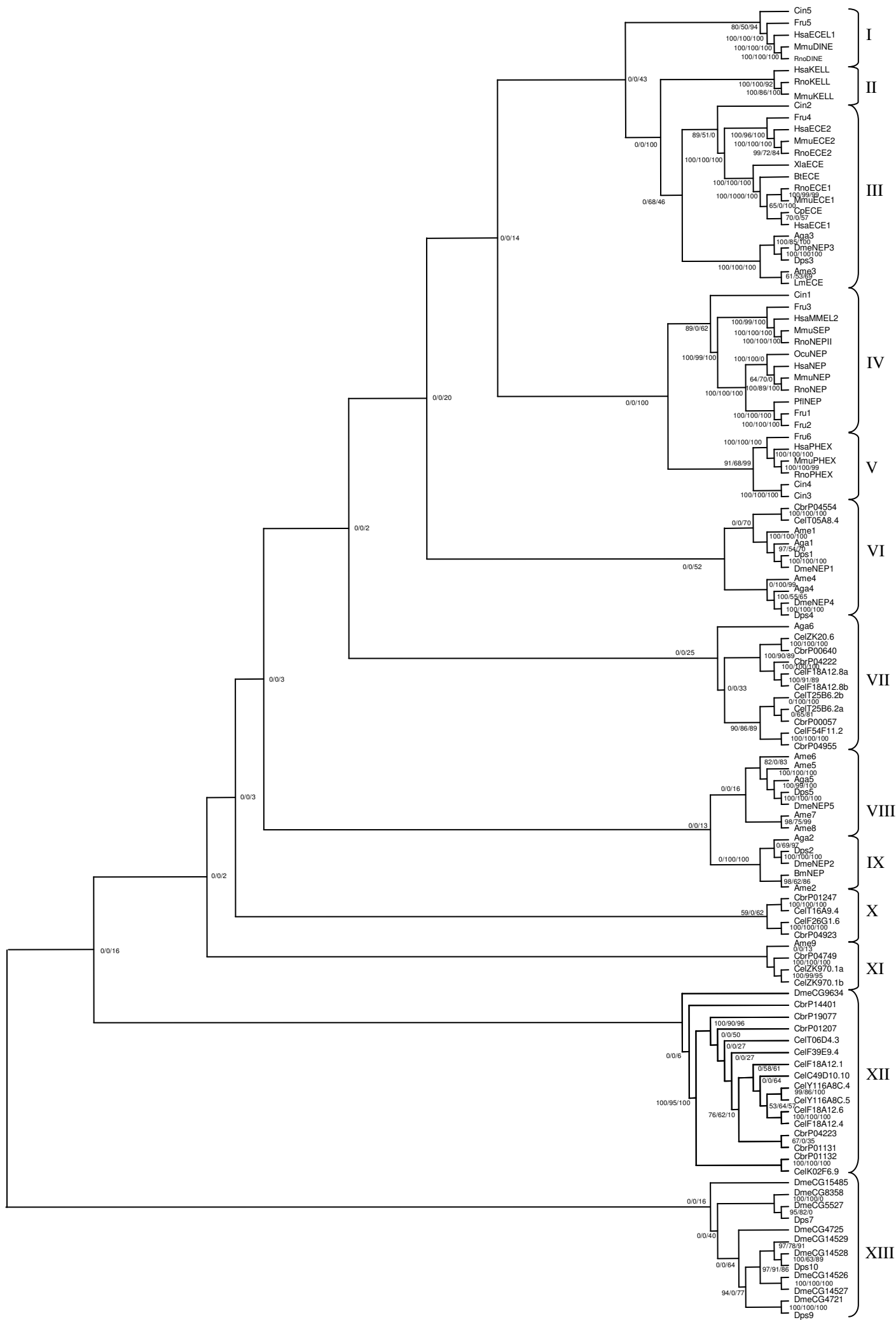

Supplement: Additional file 3 — Consensus tree of phylogenetic analyses of M13 peptidases. A majority consensus cladogram of three methods of phylogenetic reconstruction of M13 proteins, including percentage bootstrap values in the order: neighbour-joining/maximum parsimony/maximum likelihood. [file 1471-2148-8-16-S3.pdf]

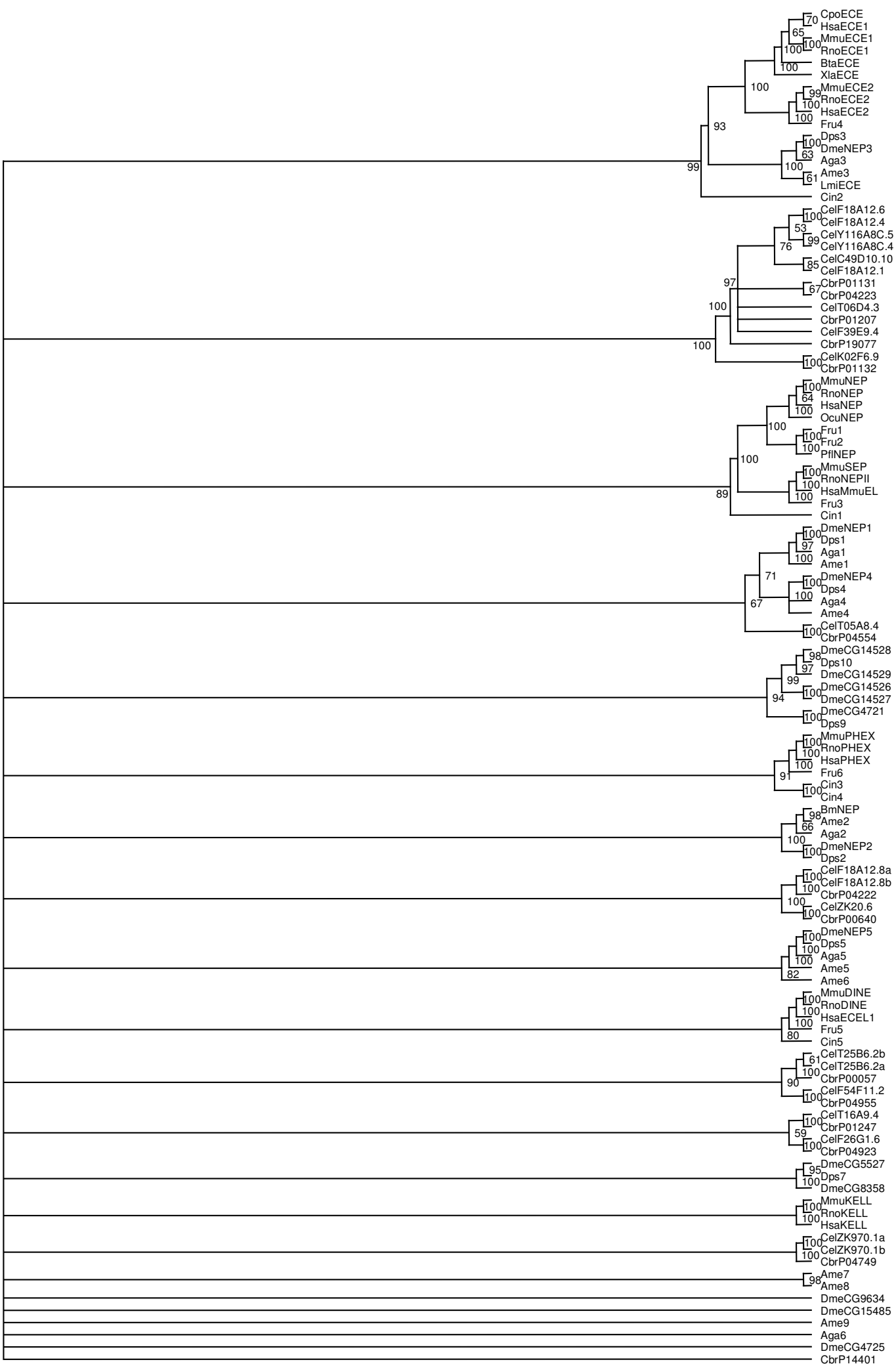

Supplement: Additional file 4 — Neighbour-joining analysis of M13 peptidases. Cladogram of neighbour-joining reconstruction of M13 proteins, including percentage bootstrap values. [file 1471-2148-8-16-S4.pdf]

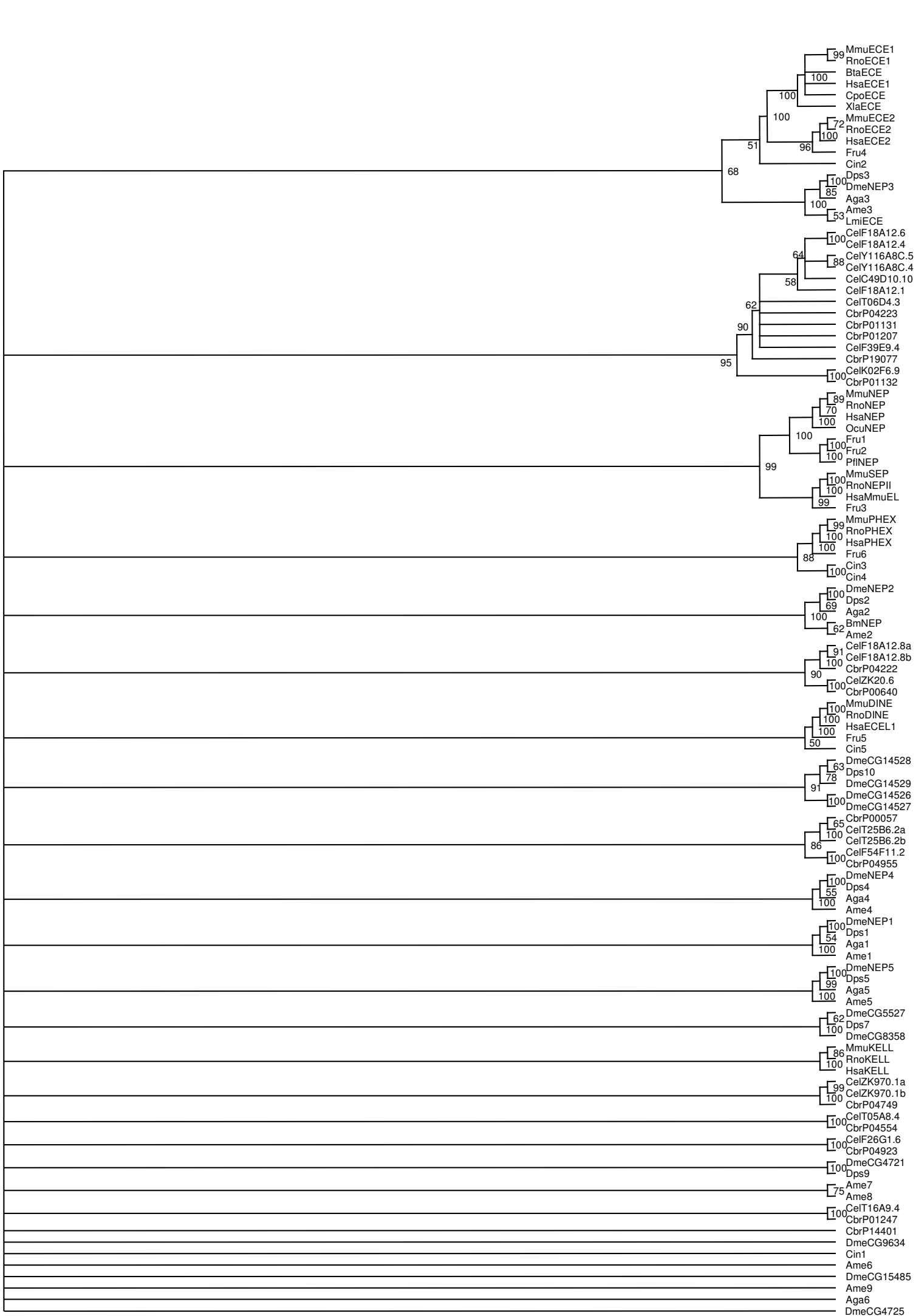

Supplement: Additional file 5 — Maximum parsimony analysis of M13 peptidases. Cladogram of maximum parsimony reconstruction of M13 proteins, including percentage bootstrap values. [file 1471-2148-8-16-S5.pdf]

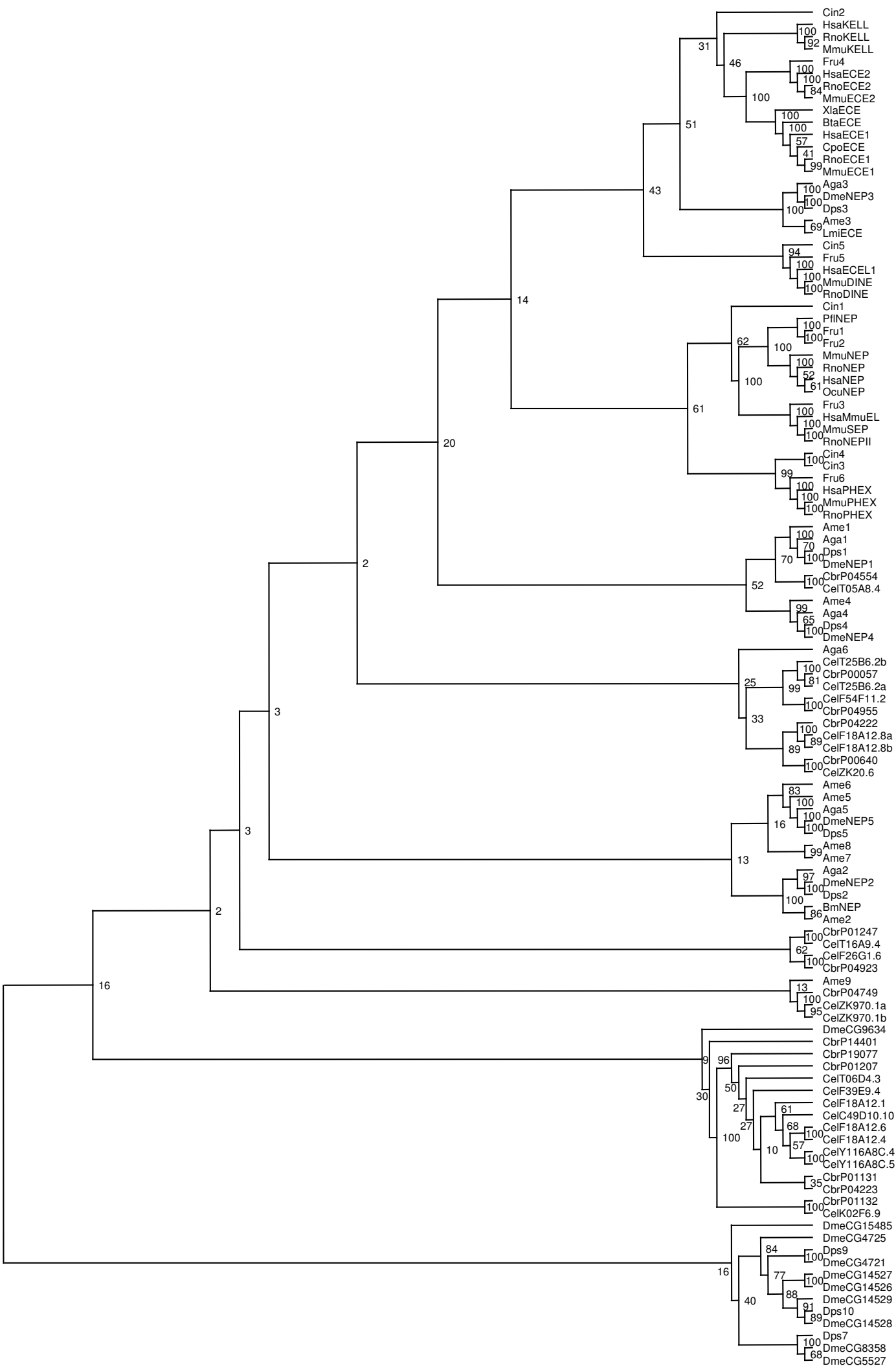

Supplement: Additional file 6 — Maximum likelihood analysis of M13 peptidases. Cladogram of maximum likelihood reconstruction of M13 proteins, including percentage bootstrap values. [file 1471-2148-8-16-S6.pdf]
